# Supplementary material for: Content-rich biological network constructed by mining PubMed abstracts
Source: BMC Bioinformatics. 2004 Oct 8;5:147. doi: 10.1186/1471-2105-5-147 (PMC528731; doi:10.1186/1471-2105-5-147)
Supplement: Additional File 2 — The original results of the above study (non-essential files are deleted to keep the file size under the limit set by BMC bioinformatics). [file 1471-2105-5-147-S2.bz2 › chilibotAdditionalFile2/dip10/396ID10775655E9950/html/EGFR_TGFA.html]

 


 **EGFR** and **TGFA** 
  
Found 1071 abstracts in PubMed, retrieved 10.  
 

 What does Google say? 
 PDF only 
| .edu only 

---

**Interactive relationship** (e.g. stimulation, inhibition, etc)

**Inhibitory relationship**- Our results demonstrate that E4T and  **TGFa**  provoke decreased Cbl recruitment,  **EGFR**  ubiquitination and  **EGFR**  degradation compared to EGF.  Ref: 12829707 J Biol Chem< MedlineTA>J Biol Chem,
- We conclude that the enhanced  **EGFR**  recycling that has been observed in HER 14 cells following  **TGFa**  or E4T stimulation correlates with decreased  **EGFR**  ubiquitination and  **EGFR**  degradation,.  Ref: 12829707 J Biol Chem< MedlineTA>J Biol Chem,
- Co treating PC 3 cells with quercetin significantly attenuated EGF and TGF alpha  [ **TGFA** ]  induced growth and phosphorylation of ErbB  [ **EGFR** ]  2, ErbB  [ **EGFR** ]  3, c Raf, MAPK kinase 1 2 MEK1 2, MAPK, Elk 1 and Akt 1.  Ref: 12888923 Int J Oncol, 2003
**Neutral relationship**- In contrast, the amount of TGF alpha  [ **TGFA** ]  bound to  **EGFR**  is found to be higher in the gastric mucosa of aged than in young rats.  Ref: 12772780 Dig Dis Sci, 2003
- Our simulations predict that when  **EGFR**  is activated with TGF alpha  [ **TGFA** ] , receptor activation is biased toward the cell surface whereas EGF produces a signaling bias toward the endosomal compartment.  Ref: 12885624 Biophys J, 2003

**Non-interactive relationship** (e.g. studied together, co-existance, homology, etc.)

- Similarly,  **EGFR**  TK inhibition by ZD1839 reversed the invasive phenotype promoted by the constitutively activated form of the  **EGFR**  EGFRvIII and the  **EGFR**  agonists transforming growth factor alpha  [ **TGFA** ]  TGFalpha, amphiregulin and EGF.  Ref: 12881705 Oncogene, 2003
- Immunohistochemical procedures were performed to detect the following protein antigens CD99, interleukin IL 1alpha, IL 6, transforming growth factor TGF alpha  [ **TGFA** ] , conventional c protein kinase C cPKC alpha, cPKC betaII, phosphorylated p PKC alpha betaII, c kit CD117, platelet derived growth factor receptor PDGFR alpha, PDGFR beta, epidermal growth factor receptor  [ **EGFR** ]   **EGFR** , human epidermal growth factor receptor  [ **EGFR** ]  HER 2 neu, cathepsin D, angiotensin converting enzyme ACE, angiotensin II type 1 AT1 receptor, p21ras, the alpha subunit of farnesyl and geranylgeranyl transferase FTalpha GGTalpha, phospho p c Jun N terminal kinase p JNK, p p38 mitogen activated protein kinase MAPK, cyclin D1, c Jun, Ki 67, bcl 2, TGF beta1 latency associated peptide LAP, TGF betaRII, and cyclooxygenase COX 2.  Ref: 12817616 Ann Clin Lab Sci< MedlineTA>Ann Clin Lab Sci, 2003
- Detection of transforming growth factor alpha  [ **TGFA** ]  and epidermal growth factor receptor  [ **EGFR** ]  mRNA and immunohistochemical localization of their proteins in the ovine uterus during the early implantation period.  Ref: 12814185 Histochem J< MedlineTA>Histochem J, 2003
- Inhibition of ErbB  [ **EGFR** ]  2 and ErbB  [ **EGFR** ]  3 expression by quercetin prevents transforming growth factor alpha  [ **TGFA** ]  TGF alpha  [ **TGFA** ]  and epidermal growth factor EGF induced human PC 3 prostate cancer cell proliferation.  Ref: 12888923 Int J Oncol, 2003
- RT PCR transcripts of  **EGFR**  ligands EGF, TGF alpha  [ **TGFA** ] , HB EGF, and amphiregulin were detected in both carcinomas and normal mucosa, indicating that autocrine growth stimulation of colorectal carcinomas is mediated by coexpression of EGF receptor ligands and upregulation of EGF receptors.  Ref: 12790326 Recent Results Cancer Res, 2003
- Here we evaluated  **EGFR**  activation, Cbl recruitment,  **EGFR**  ubiquitination and degradation in response to EGF,  **TGFa**  and E4T.  Ref: 12829707 J Biol Chem< MedlineTA>J Biol Chem, 2003
- To determine the utility of this approach, we simulated the differential activation of the  **EGFR**  by two of its ligands, epidermal growth factor EGF and transforming growth factor alpha  [ **TGFA** ]  TGF alpha  [ **TGFA** ] .  Ref: 12885624 Biophys J, 2003
